# Alpha-Gal Syndrome: An Emerging Tick-Borne Allergy to Red Meat

**DOI:** 10.7759/cureus.79746

**Published:** 2025-02-27

**Authors:** Tyanna McCladdie, Michael Herman

**Affiliations:** 1 Osteopathic Medicine, Philadelphia College of Osteopathic Medicine, Moultrie, USA; 2 Gastroenterology, Borland Groover, Jacksonville, USA

**Keywords:** alpha gal, alpha-gal syndrome, lone star tick, rare allergy, red meat, red meat allergy, tick-bite

## Abstract

Alpha-gal syndrome (AGS) is a delayed-onset food allergy triggered by an immune response to galactose-α-1,3-galactose (alpha-gal), a carbohydrate associated with Lone Star tick bites. A 45-year-old female presented with a 9-month history of nausea and abdominal pain consistently associated with red meat consumption. Initial workup, including routine labs, imaging, and endoscopy, was unremarkable, but specific IgE testing confirmed AGS. Management included strict avoidance of red meat, carrying an epinephrine auto-injector, and referral to an allergist for education and long-term care. This case highlights the diagnostic challenges posed by the delayed reaction and the importance of dietary vigilance and emergency preparedness. Increased awareness of AGS is essential, especially in regions where tick exposure is prevalent.

## Introduction

Alpha-gal syndrome (AGS) is a unique, delayed-onset food allergy triggered by an immune response to galactose-α-1,3-galactose (alpha-gal), a carbohydrate found in the tissues of non-primate mammals, commonly referred to as red meat. AGS was first discovered by researchers investigating anaphylactic reactions in patients treated with cetuximab, a monoclonal antibody used to treat colorectal cancer and squamous cell carcinoma of the head and neck [1, 2, 3]. They identified that patients who had a reaction to cetuximab infusions also had IgE-specific antibodies to an oligosaccharide, alpha-gal, on the fragment antigen-binding region of cetuximab [1-3]. It was later found that the IgE-specific antibodies to alpha-gal were linked to bites from ticks, which is most often the Lone Star tick (*Amblyomma americanum*) in the United States. AGS has since been discovered outside the United States in Europe, Australia, and parts of Asia [2]. These bites are believed to sensitize individuals by introducing alpha-gal through tick saliva, leading to the production of IgE antibodies [1]. Some patients develop urticaria at the location of previous tick bites after consuming red meat, suggesting a localized memory response to alpha-gal [4].

Unlike most food allergies which involve immediate hypersensitivity reactions to proteins, AGS presents a distinct pathophysiology with reactions occurring hours after exposure to alpha-gal-containing products. This delay complicates diagnosis, as patients and healthcare providers might not immediately connect the symptoms to red meat consumption. The symptoms of AGS can range from mild gastrointestinal discomfort to severe anaphylaxis, often complicating diagnosis and management [1]. Gastrointestinal symptoms, such as nausea, vomiting, and abdominal pain, are common initial presentations, and this variability in symptoms can lead to misdiagnosis or delayed diagnosis. The diagnosis of AGS requires a detailed patient history including the onset and timing of symptoms and identifying associated factors. The diagnosis can be confirmed by laboratory blood tests that measure specific IgE antibodies to alpha-gal. A positive test result for alpha-gal IgE antibodies is characterized as $\ge$ 0.1 kU/L [5]. However, not all patients with AGS will have detectable levels of alpha-gal-specific IgE, which can further complicate the diagnostic process [1, 2].

The increasing recognition of AGS, particularly in endemic areas, underscores the need for awareness among healthcare providers. This is critical in the southeastern United States where tick populations are growing and human-tick encounters are becoming more common [2]. Increased awareness and understanding of AGS will aid in quicker diagnosis, effective management, and better patient outcomes. This case report aims to highlight the diagnostic challenges and clinical management of a patient with AGS, adding to the growing body of literature on this emerging allergy.

## Case presentation

A 45-year-old female with a past medical history of hypothyroidism that is well-controlled on hormone replacement therapy, presented with a 9-month history of nausea and abdominal pain typically 3-4 hours after eating. The symptoms were consistently associated with the consumption of red meat and did not improve despite the exclusion of other common dietary triggers. She denied any associated symptoms such as rash, dyspnea, or syncope. There was no personal or family history of atopy, food allergies, or other allergic conditions. The patient reported frequent outdoor activities, including camping trips with her family in North Florida, where she often spent time in wooded areas. This history raised the suspicion of tick exposure as a potential trigger for her symptoms. 

Initial laboratory evaluations, including a complete metabolic panel (CMP), complete blood count (CBC), thyroid-stimulating hormone (TSH), and erythrocyte sedimentation rate (ESR), were within normal limits, as listed in Table 1. Further diagnostic workup included a small bowel follow-through (SBFT), hepatobiliary scan, and esophagogastroduodenoscopy (EGD), all of which were unremarkable. Given the clinical suspicion of AGS, specific IgE testing for alpha-gal antibodies was ordered. The results revealed an alpha-gal IgE level of 0.2 kU/L, consistent with sensitization to alpha-gal and supporting the diagnosis of AGS.

**Table 1:** Laboratory values K/mcL: thousands per microliter; g/dL: grams per deciliter; mmol/L: millimoles per liter; mg/dL: milligrams per deciliter; U/L: units per liter; mIU/L: milli-international units per liter; mm/hr: millimeters per hour; mg/L: milligrams per liter

Laboratory Test	Value	Reference Range
White blood cells	5.8 K/mcL	4.0 - 11.0 K/mcL
Hemoglobin	13.5 g/dL	12.0 - 16.0 g/dL
Hematocrit	40.2%	37.0 - 47.0%
Platelet count	195 K/mcL	150 - 400 K/mcL
Neutrophils	58%	50 - 70%
Lymphocytes	32%	20 - 40%
Eosinophils	5.5%	1 - 4%
Sodium	138 mmol/L	135 - 145 mmol/L
Potassium	4.1 mmol/L	3.5 - 5.1 mmol/L
Chloride	102 mmol/L	98 - 107 mmol/L
Carbon dioxide (CO₂)	24 mmol/L	22 - 29 mmol/L
Blood urea nitrogen (BUN)	13 mg/dL	7 - 20 mg/dL
Creatinine	0.89 mg/dL	0.6 - 1.2 mg/dL
Glucose	92 mg/dL	70 - 100 mg/dL
Calcium	9.4 mg/dL	8.6 - 10.3 mg/dL
Total Protein	7.1 g/dL	6.0 - 8.3 g/dL
Albumin	4.2 g/dL	3.5 - 5.0 g/dL
Aspartate aminotransferase (AST)	42 U/L	10 - 40 U/L
Alanine aminotransferase (ALT)	45 U/L	7 - 56 U/L
Alkaline phosphatase	72 U/L	44 - 120 U/L
Total bilirubin	0.8 mg/dL	0.1 - 1.2 mg/dL
Thyroid-stimulating hormone (TSH)	2.3 mIU/L	0.4 - 4.5 mIU/L
Erythrocyte sedimentation rate (ESR)	22 mm/hr	< 20 mm/hr
C-reactive protein (CRP)	3.1 mg/L	< 5.0 mg/L

## Discussion

The patient’s symptoms and history align with AGS, characterized by a delayed reaction to red meat and other mammalian-derived products. Regional tick exposure plays a pivotal role in sensitizing individuals to alpha-gal, emphasizing the importance of considering geographic factors in diagnosis and management. The typical presentation of AGS often includes urticaria, angioedema, or anaphylaxis in addition to gastrointestinal symptoms. An example of a typical presentation was seen in a case report by Heffes-Doon et al., where the patient presented with a pruritic rash on his extremities and angioedema hours after consuming mammalian meat [6]. Unlike the typical presentation, our patient experienced only gastrointestinal symptoms such as nausea and abdominal pain. The atypical presentation seen in this patient is consistent with other reports that highlight the diverse clinical manifestations of AGS. A study by Commins et al. noted that gastrointestinal symptoms are common in AGS, often leading to misdiagnosis or delayed diagnosis due to their nonspecific nature [1, 2]. The absence of a clear atopic history further complicates the diagnosis, as AGS can manifest in individuals without pre-existing allergies [7]. This case highlights the variability in AGS presentations, where not all patients exhibit the hallmark allergic reactions [8]. 

Several factors may explain our patient’s atypical presentation. First, her level of sensitization, as measured by alpha-gal-specific IgE antibodies, may have been sufficient to cause symptoms but not severe enough to trigger anaphylaxis or systemic allergic reactions. Additionally, the amount of alpha-gal exposure during meals could have played a role, as smaller doses are less likely to provoke a significant immune response [2]. Co-factors such as exercise, alcohol, or medications like NSAIDs, which can exacerbate immune responses in AGS, were absent in this case, potentially contributing to the absence of a severe reaction [9]. The delay in symptom onset, typically occurring 3-6 hours after ingestion, further complicates the clinical picture, as gastrointestinal symptoms alone may be attributed to other causes without a high index of suspicion [8]. In another case of atypical presentation of AGS, the patient originally presented with just gastrointestinal symptoms and later developed a widespread rash, further adding to the complexity of this disease presentation [10]. 

In summary, the patient’s lifestyle, including frequent outdoor activities and camping trips in North Florida, placed her at higher risk of exposure to Lone Star ticks, likely sensitizing her to alpha-gal [1, 2]. Despite the absence of anaphylaxis or rash, her consistent symptoms with red meat consumption and positive alpha-gal-specific IgE testing confirmed the diagnosis. This case underscores the importance of recognizing non-classic presentations of AGS, especially in endemic regions. Management focused on strict dietary avoidance of red meat and related products, as well as carrying an epinephrine auto-injector for emergency use, should symptoms escalate. Ongoing education on tick bite prevention and dietary vigilance remains critical to reducing the risk of future sensitization or reactions [2, 9].

## Conclusions

This case highlights the complexity and variability of AGS. This patient, who presented with gastrointestinal symptoms rather than classic allergic reactions, underscores the importance of considering AGS in individuals with unexplained symptoms linked to red meat consumption, especially in endemic areas. Diagnosis requires a detailed clinical history, recognition of risk factors such as tick exposure, and confirmatory IgE testing. Management is centered on dietary avoidance, patient education, and emergency preparedness, with a comprehensive approach to ensure optimal outcomes. As the range of the Lone Star tick continues to expand, increasing awareness of AGS among healthcare providers and the public is essential to facilitate timely diagnosis and effective management, improving the quality of life for affected individuals.
